# Pearls and pitfalls of optical coherence tomography angiography in the multimodal evaluation of uveitis

**DOI:** 10.1186/s12348-017-0138-z

**Published:** 2017-10-05

**Authors:** Francesco Pichi, David Sarraf, Mariachiara Morara, Shahana Mazumdar, Piergiorgio Neri, Vishali Gupta

**Affiliations:** 1 Eye Institute, Cleveland Clinic Abu Dhabi, Al Maryah Island, PO Box 112412, Abu Dhabi, United Arab Emirates; 2 Cleveland Clinic Lerner College of Medicine, Case Western Reserve University, Cleveland, USA; 3 Stein Eye Institute, University of California, Los Angeles, Los Angeles, CA USA; 4 Greater Los Angeles VA Healthcare Center, Los Angeles, CA USA; 5 Eye Clinic, Policlinico Sant’Orsola-Malpighi, Bologna, Italy; 6 ICARE eye hospital and Post Graduate Institute, Noida, India; 7 The Ocular Immunology Service, The Eye Clinic, Polytechnic University of Marche, Ancona, Italy; 8 Advanced Eye Centre, Post Graduate Institute of Medical Education and Research, Chandigarh, India

**Keywords:** Optical coherence tomography angiography, Uveitis, Fluorescein angiography, Indocyanine green angiography

## Abstract

**Background:**

Optical coherence tomography angiography (OCTA) employs a novel imaging algorithm that detects the amplitude or phase decorrelation of blood cell movement. It thus provides a flow map with depth-resolved visualization of the various vascular layers in the posterior pole of the eye including the retina capillary plexus and the choroid.

In the past 3 years, the number of research papers on the subject of OCTA in retinal diseases has grown exponentially including important applications in the field of uveitis. While the study of OCTA in uveitic diseases has gained remarkable relevance worldwide, interpretation can be challenging, and many limitations exist in optimally using this advanced system in uveitic eyes.

The aim of this review is to describe the many significant applications of OCTA in uveitis disorders and to outline the various limitations that can confound interpretation and support uveitis specialists in the integration of OCTA in the multimodal imaging approach to inflammatory diseases.

**Main body:**

Unlike conventional angiography that can dynamically detect inflammation and leakage of dye from retinal vessels, OCTA provides other important biomarkers of inflammation. Detailed microvascular reconstruction of normal and abnormal blood vessels and quantitative evaluation are advantages of OCTA analysis. OCTA can therefore non-invasively detect choroidal neovascularization that may complicate inflammatory disorders, and with remarkable depth-resolved capability, OCTA can identify and quantitate flow loss as a manifestation of ischemia and/or inflammation.

The areas of flow deficit on OCTA at the level of the inner choroid often co-localize with hypofluorescent lesions with indocyanine green angiography. These regions of presumed choriocapillaris ischemia may occur in placoid disorders. Space-occupying granulomas may occur in disorders such as sarcoid and may or may not co-localize with choriocapillaris ischemia on ICG angiography. Blocking or shadowing artifacts should be excluded when evaluating inner choroidal abnormalities with OCT angiography.

Fundus autofluorescence may assess the metabolic function of the retinal pigment epithelium (RPE) and the viability of the overlying photoreceptors and thus the activity of inflammation associated with uveitic lesions. The photoreceptors are physiologically maintained by the diffusion of oxygen from the choriocapillaris below and, to a lesser extent, from the deep retinal capillary plexus above. The depth-resolved capability of OCTA may therefore provide additional significant microvascular information about these vascular layers that may be driving the development of hyper-autofluorescent RPE inflammation and photoreceptor loss.

**Conclusions:**

The implementation of OCTA in the evaluation and management of uveitis disorders is being spurred by our greater knowledge and understanding of its application. In order to take full advantage of this exciting new imaging modality, however, uveitis specialists must understand the limitations of interpretation and potential artifact-related pitfalls in assessment and should continue to support evaluation with multimodal imaging to best optimize diagnoses and treatment of inflammatory diseases.

## Review

The evolution of optical coherence tomography (OCT) over the last two decades has been remarkable for a sustained improvement in the resolution and sensitivity of image detection. OCT has also provided the platform for the development of other significant imaging advancements including optical coherence tomography angiography (OCTA) [1]. This is an innovative technique that employs amplitude or phase decorrelation to detect blood flow without the need for intravenous dye administration [2]. The past 3 years have witnessed an explosion in the application of this imaging method in neovascular diseases, and recently, more and more studies are being published on the subject of OCTA in uveitis [3]. OCTA may provide an important tool in the evaluation of inflammatory eye diseases as vascular changes in the iris, choroid, and retina play an important role in the pathophysiology of ocular inflammation.

Vascular abnormalities in uveitis may be caused by various pathological processes including vascular occlusion, local ischemia, and alteration of cell mediators. OCTA can be used to accurately detect vascular flow changes and neovascularization and may provide objective, qualitative, or quantitative inflammatory biomarkers. In addition, OCTA image acquisition is fast and easy and non-invasive, limiting the risk of adverse events for the patient. Depth-resolved capability provides important evaluation of the deep retinal capillary plexus that may be selectively targeted in retinal vascular or inflammatory disease. However, the importance of a multimodal approach is recommended because of the significance of dye leakage from inflamed vessels which can provide an additional important marker of inflammation [4, 5].

The purpose of this review is to summarize the current applications of OCTA in the evaluation and management of uveitic retinal and choroidal disorders, with emphasis on a multimodal approach to include wide-field dye-based angiography to evaluate inflammatory disorders such as retinal vasculitis, and indocyanine angiography (ICGA) to evaluate choroidal ischemia in eyes with placoid variant disorders.

### Fluorescein angiography versus optical coherence tomography angiography

During the procedure of fluorescein angiography (FA), fluorescein sodium, a natural dye substance, is injected intravenously in order to analyze the retinal vascular circulation in the posterior pole and periphery [6]. Fluorescein is a small hydrosoluble molecule of 354 Da of which 80% is bound to proteins and 20% is free. The emission fluorescence is captured with a filter set at 520–530 nm within the wavelengths of visible light. The retinal vessels do not normally leak dye and are therefore well visualized with FA. However, the inner choroid is poorly resolved due to widespread leakage from the fenestrated choriocapillaris which obstructs the larger deeper choroidal vessels and which obstructs the details of the choriocapillaris, although the lobular morphology can be identified during the early phases of the angiogram.

Fluorescein has micromolecular properties and may easily leak with the slightest disruption of the blood-retinal barrier. Retinal vessels are highly impermeable due to the tight junctions between endothelial cells and do not normally leak. However, even mild inflammation of the retinal vessel wall may result in retinal vascular leakage [7], and therefore, FA is a very sensitive way to detect retinal vessel inflammation. Vascular leakage on FA can be essential in assessing inflammatory activity.

**Fig. 1:**
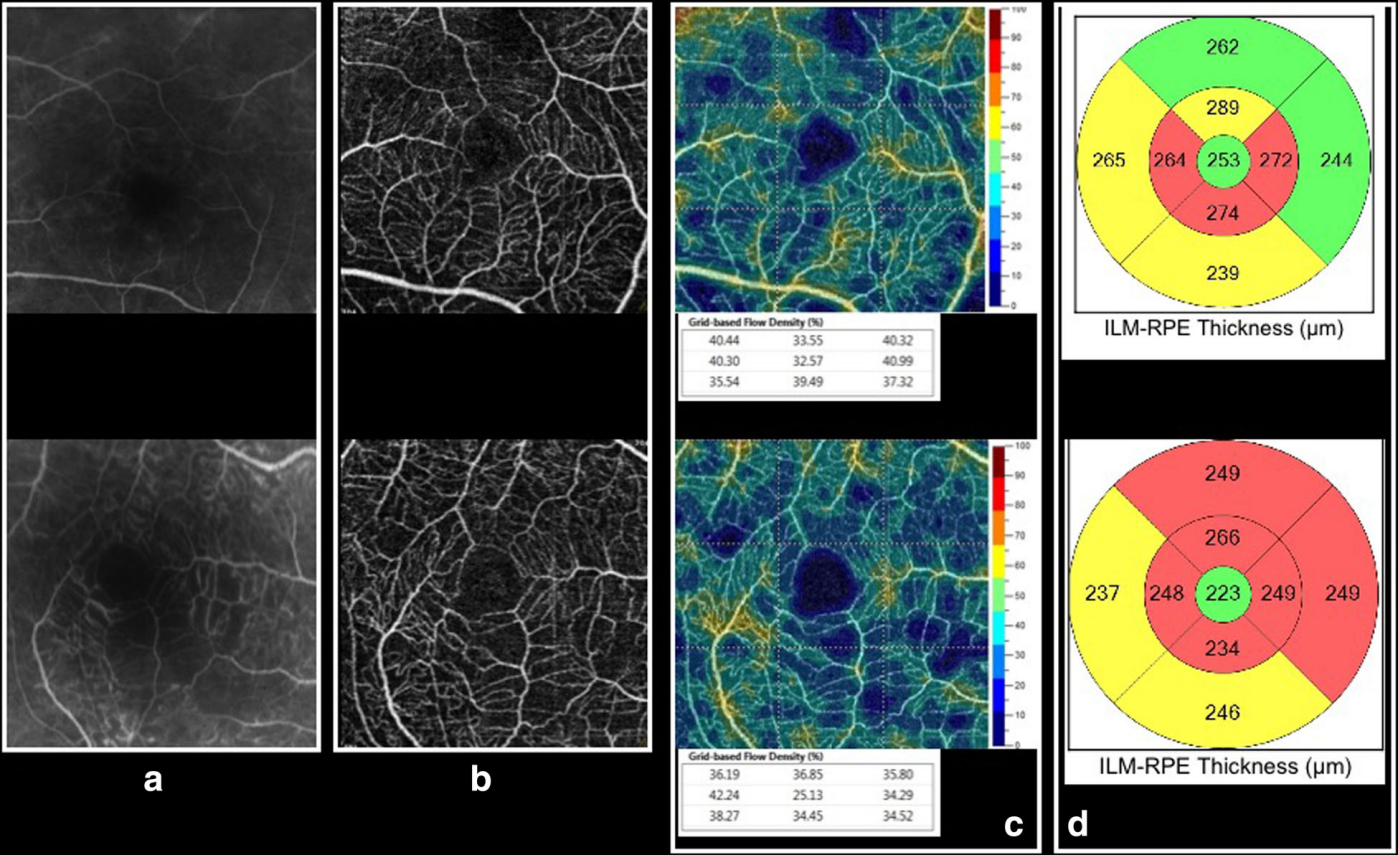
Comparison of fluorescein angiography and OCTA in posterior uveitis. Two eyes of two patients with chronic birdshot chorioretinopathy illustrate mild leakage on fluorescein angiography (**a**) that masks or blocks areas of reduced flow. OCTA more accurately highlights the zones of non-perfusion in the superficial retinal capillary plexus (**b**). Note the generalized reduction in blood flow velocity (highlighted in blue) that is evident in the color-coded OCTA map (**c**) that can also provide quantitative parameters. The areas of flow reduction on OCTA corresponds to a thinning of the retina on the OCT map (**d**) that has been described in the literature in advanced stages of birdshot

While OCTA may not detect leakage, it can illustrate selective changes in the density of vessels in the superficial and/or deep retinal capillary plexus in patients with vasculitis (Fig. 1), as illustrated in two studies [8, 9]. Employing a prototype SD-OCTA device (Cirrus, Carl Zeiss Meditec, Inc., Dublin, CA, USA), Kim et al [8] performed cross-sectional OCTA analysis of 28 eyes with retinal vasculitis. A significantly lower parafoveal capillary density was illustrated in the superficial retinal plexus of uveitic compared to healthy eyes. Bessete et al. [9] employed 3 × 3 mm and 6 × 6 mm OCT angiography (Avanti RTVue-XR, Optovue Inc., Fremont, CA) in 26 patients (52 eyes) with retinal vasculitis and identified similar abnormalities including flow deficits in the superficial retinal capillary plexus and enlargement and/or irregularity of the foveal avascular zone and capillary microvascular remodeling but normal flow in the presence of exudates. The highly significant differences in capillary density and morphology that was detected in these two vasculitis studies illustrate that the effects of intraocular inflammation can be quantitatively measured within a 3 × 3 mm parafoveal window using OCTA.

The importance of depth-resolved capability with OCTA to assess the deep vascular complex cannot be underestimated. Conventional dye-based angiography poorly visualizes the deeper capillary layers. Recent studies have identified more significant capillary flow deficit in the deep retinal capillary plexus using OCTA in diseases such as birdshot chorioretinopathy. Assessing the flow density of the deep vascular complex may provide more complete information regarding the deleterious effects of inflammation and may provide an additional biomarker of inflammatory activity that may be quantified to assess progression and response to therapy.

This reduction in retinal capillary flow does not necessarily mean vascular obliteration or non-perfusion. Leakage of plasma outside the inflamed wall may lead to a decrease in the flow velocity inside the retinal vessel. In fact, OCTA may not detect retinal capillary flow outside the range of 0.3 mm/s to 2 mm/s. Thus, in active vasculitis with FA leakage, there may be a global slowdown of the retinal circulation that may be identified with OCTA. Future studies are necessary to demonstrate a correlation between fluorescein leakage and the decrease in the capillary flow on OCTA.

Leakage of dye in posterior uveitis can limit our ability to evaluate adjacent capillary perfusion and can be present due to damaged or ischemic capillary beds. In this case, the inability of OCTA to detect leakage is an advantage and can provide microvascular morphological detail and information regarding capillary perfusion, with quantitative capability, in both the superficial and deep capillary plexus. In patients with reperfusion of a prior branch retinal artery occlusion, for example, capillary dropout is more easily identified with OCTA compared to FA and, in some cases, not even visible with FA. As such, the absence of vascular leakage with OCTA can be advantageous as hyperfluorescence due to vascular leakage, or window defects may impede the detection of capillary perfusion.

FA is currently the gold standard for identifying inflammatory CNV [10]. In various uveitic choroidal disorders such as multifocal choroiditis (MFC) or punctate inner choroidopathy (PIC), the major cause of vision loss may be related to direct inflammatory damage of the retina and RPE [11], and secondary invasion of inflammatory CNV [12]. Making the distinction between inflammatory versus neovascular lesions is mandatory to optimize therapy. Both illustrate elevation of the RPE by homogeneous hyper-reflective material with SD-OCT analysis [3, 13]. When avascular, these inflammatory lesions may appear small and conical [3]. It is unclear exactly what comprises the sub-RPE material. Histopathologic studies [14] of these inflammatory lesions are sparse but have reported the presence of choroidal infiltrates with B-cell lymphocytes. In the case of inflammatory lesions, the disruption of the choroid [14] occurs because of cytokines and the physical dehiscence in the RPE. More heterogenous subretinal material, wider in diameter [15], may indicate the presence of CNV that can rupture through the RPE and lead to exudation in the subretinal space.

Inflammation and CNV are not mutually exclusive and may both be present in certain lesions. Even with multimodal imaging, the differentiation between active inflammatory lesions and inflammatory CNV may not be possible because both have the potential to cause infiltration and exudation with breakdown in the blood–retina barrier.

FA is currently the gold standard for identifying CNV [16]. The typical presentation of inflammatory CNV on FA is early hyperfluorescence with late leakage [10], while inflammation displays early hypofluorescence or isofluorescence with late hyperfluoresence [10]. However, CNV may not always illustrate obvious early hyperfluorescence due to blockage from the inflammatory component, fluid, or hemorrhage. Furthermore, inflammatory lesions may display early hyperfluorescence because of RPE damage causing a window defect.

**Fig. 2:**
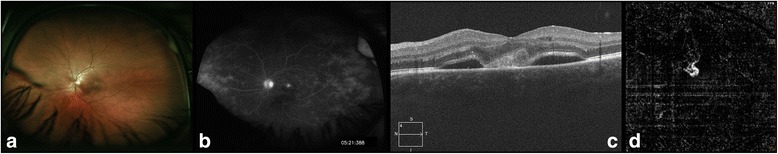
Inflammatory choroidal neovascular membrane. The left eye of a patient with posterior uveitis (**a**) does not show retinal or choroidal lesions on fundus photography, but RPE mottling in the foveal area. Fluorescein angiography illustrates retinal vascular leakage (**b**) in the extreme periphery and an area of focal leakage superior to the fovea. This corresponds to subretinal hyperreflective material (SHRM) on the spectral domain OCT (**c**) and a type 2 choroidal neovascular membrane surrounded by sub-retinal fluid. The CNV is clearly identified in the outer retina on OCTA (**d**)

It is therefore challenging to distinguish inflammatory CNV from purely inflammatory lesions by FA. OCTA, however, has improved the sensitivity and specificity of CNV detection in inflammatory choroidal disorders. The microvascular morphological detail may be more precisely detected with OCTA, and the area and the density of the inflammatory CNV can be quantitatively measured. OCTA has shown remarkable accuracy in distinguishing inflammatory CNV (Fig. 2) from avascular inflammatory lesions that were poorly identified using other imaging modalities [3, 17]. However, OCTA cannot reliably determine which lesions are clinically active [12].

### Indocyanine green angiography and optical coherence tomography angiography

Indocyanine green (ICG) fluoresces at 830 nm, and therefore, ICG angiography (ICGA) provides better visualization of the choroidal vascular structures through the retinal pigment epithelium [18]. ICG is nearly completely albumin protein bound, and as such, imaging of the deeper choroidal vessels is not obstructed by significant choriocapillaris leakage as with FA [19]. In the retina, only major damage to retinal vessels will result in ICG leakage. In the choroid, however, ICG may leak very slowly from the fenestrated choriocapillaris. During recirculation, more and more ICG is entrapped in the choroidal tissue as the ICG-protein complex is only slowly reabsorbed into the circulation. Gradual staining of the choroid occurs with time, causing intermediate and late choroidal background fluorescence [20].

ICG hyperfluorescence is believed to result from two mechanisms [18] in inflammatory disorders of the fundus, and therefore, choroiditis has been divided into two broad categories, choriocapillaritis versus stromal choroiditis [20]. Inflammatory choriocapillaritis may lead to non-perfusion, identified early with dye-based angiography, and irregular areas of hypofluorescent staining. This ICG pattern is best displayed in placoid disorders including acute posterior multifocal placoid pigment epitheliopathy (APMPPE) and serpiginous choroiditis (SC).

**Fig. 3:**
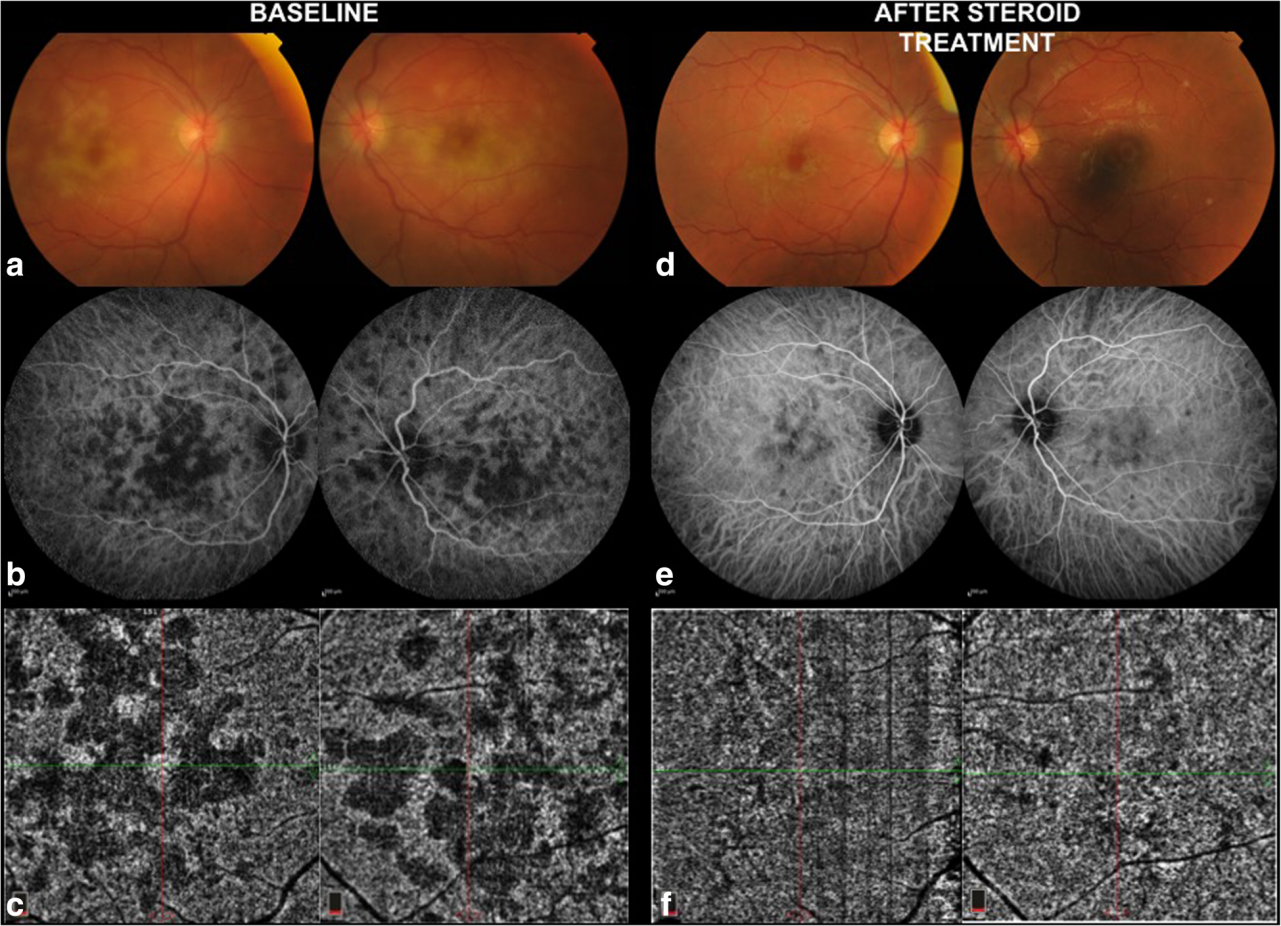
Right and left eye of a patient with APMPPE. At baseline, the creamy-white lesions (**a**) correspond to hypofluorescent areas on ICGA (**b**). These prove to be true areas of choriocapillaris ischemia on OCTA (**c**) that precisely co-localize with the shape and pattern of the ICGA lesions. After 3 weeks of oral steroids, the lesions disappear on fundus photography (**d**). Mild choroidal hypoperfusion is still detected in ICGA (**e**) and OCTA (**f**) but with a remarkable improvement compared to baseline

Controversy has persisted regarding the pathogenesis of APMPPE since its initial description by Gass [21], who chose the term “pigment epitheliopathy” to reflect the most significant clinical tissue involvement. However, the advent of ICGA in the 1990s has provided additional insights into APMPPE and has supported choroidal involvement as a prominent feature [22]. Still, several features of APMPPE remained atypical for a choroiditis including the rapid resolution of lesions and the near absence of damage to the choroid despite significant permanent alternations in the RPE. Klufas et al. [23] have recently studied OCTA technology to assess the choroid in the eyes with various placoid disorders including APMPPE. In their series, 96% of the APMPPE eyes illustrated evidence of inner choroidal or choriocapillaris flow reduction or ischemia on analysis of segmented OCTA scans that correlated with onset of visual symptoms. The zone of choriocapillaris ischemia as identified with OCTA (Fig. 3) was the same or greater than the area of hypofluoresence on ICGA. OCTA has therefore confirmed that ICGA hypofluorescence in APMPPE is indeed the result of reduced inner choroidal or choriocapillaris flow rather than blockage from overlying RPE changes, and may provide a non-invasive biomarker of choroidal inflammatory ischemic activity and response to therapy.

Other placid disorders include serpiginous choroiditis and serpiginous-like choroiditis, two distinct forms of choroiditis also believed to result from the inflammation of the choriocapillaris/inner choroid and the RPE [24]. During the active stage of these two diseases, OCTA of the choriocapillaris illustrates clearly demarcated areas of flow deficit that precisely co-localize with the hypofluorescent ICG lesions [25]. Point-to-point registration between the hypofluorescent areas on ICGA to the areas of flow-void demonstrated on OCTA can be precisely identified [25]. Additionally, intervening areas of preserved choriocapillaris [25, 26] and medium-sized choroidal vessels are identified between the active lesions. As the lesions heal, deeper medium-to-large choroidal vessels become more prominent and are better delineated on OCTA than on ICG.

The second main mechanism causing hypofluorescence with ICGA is the presence of inflammatory foci in the choroid [20] that can occupy space and block diffusion of the ICG molecule to these areas that appear dark against the physiologically fluorescent background of the choroidal stroma. These hypofluorescent lesions are round and evenly distributed in the choroidal stroma of inflammatory disorders such as Vogt-Koyanagi-Harada (VKH) and birdshot chorioretinopathy [27].

Early birdshot lesions are inflammatory choroidal infiltrates that consist of epithelioid cells [28] and that surround the choroidal stromal melanocytes. With ICGA, these lesions are hypofluorescent and line up along the larger choroidal vessels [27]. The active outer choroidal lesions are partial thickness and do not affect the adjacent choriocapillaris early in the course of disease and therefore cannot be initially visualized on OCTA analysis [3, 29]. Once these lesions depigment, the granulomas resolve with atrophy of the choriocapillaris and inner stroma [28]. The advanced birdshot lesions that are depigmented and atrophic with fundus photography co-localize with areas of flow reduction with OCTA due to the absence of choriocapillaris flow beneath the disrupted RPE.

**Fig. 4:**
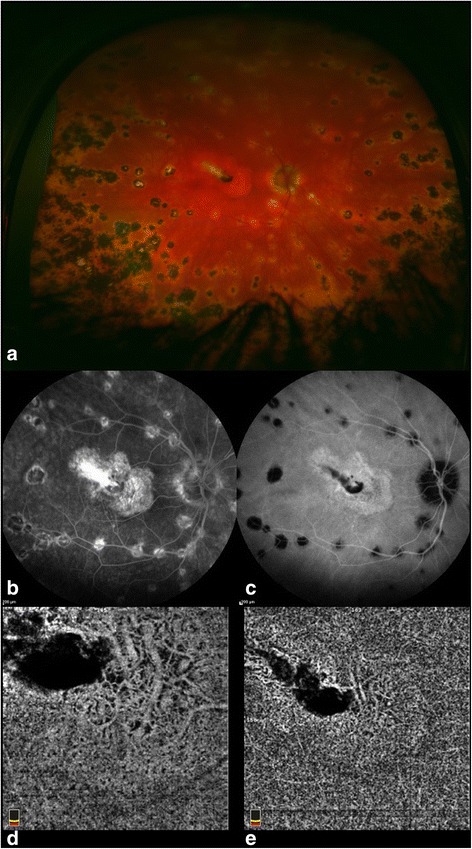
In the acute stage of VKH, fundus examination reveals small nodules at the level of the RPE, known as Dalen-Fuchs nodules, in the posterior pole (**a**). Fluorescein angiography (**b**) illustrates multifocal areas of punctate hypofluorescence with late staining or leakage and central pooling within the subretinal fluid. Indocyanine green angiography (**c**) indicates active inflammation at the level of the choroid as evidenced by the hypofluorescent dark spots. 3 × 3 mm (**d**) and 6 × 6 mm (**e**) optical coherence tomography angiography illustrates areas of flow void at the level of the choriocapillaris (**d**, **e**) proving that the choriocapillaris is involved and ischemic

Histopathologic studies have shown that while VKH disease primarily affects the choroidal stroma with diffuse infiltration of inflammatory cells, the choriocapillaris may also be involved, especially in recurrent cases, resulting in localized ischemic abnormalities [30]. The inflammation primarily involves the choroidal stroma with subsequent involvement of the RPE and outer retina. On OCTA [3], the choriocapillaris layer (below the RPE-Bruch’s membrane complex) illustrates multiple dark foci (Fig. 4) with loss of the choriocapillaris (or severe hypoperfusion) that appear as areas of flow void [31]. The edges of these areas are discrete and clearly demarcated. In comparing OCTA with ICGA, the areas of flow void on OCTA illustrate consistent co-localization with the persistent hypocyanescent spots on ICGA [31].

**Fig. 5:**
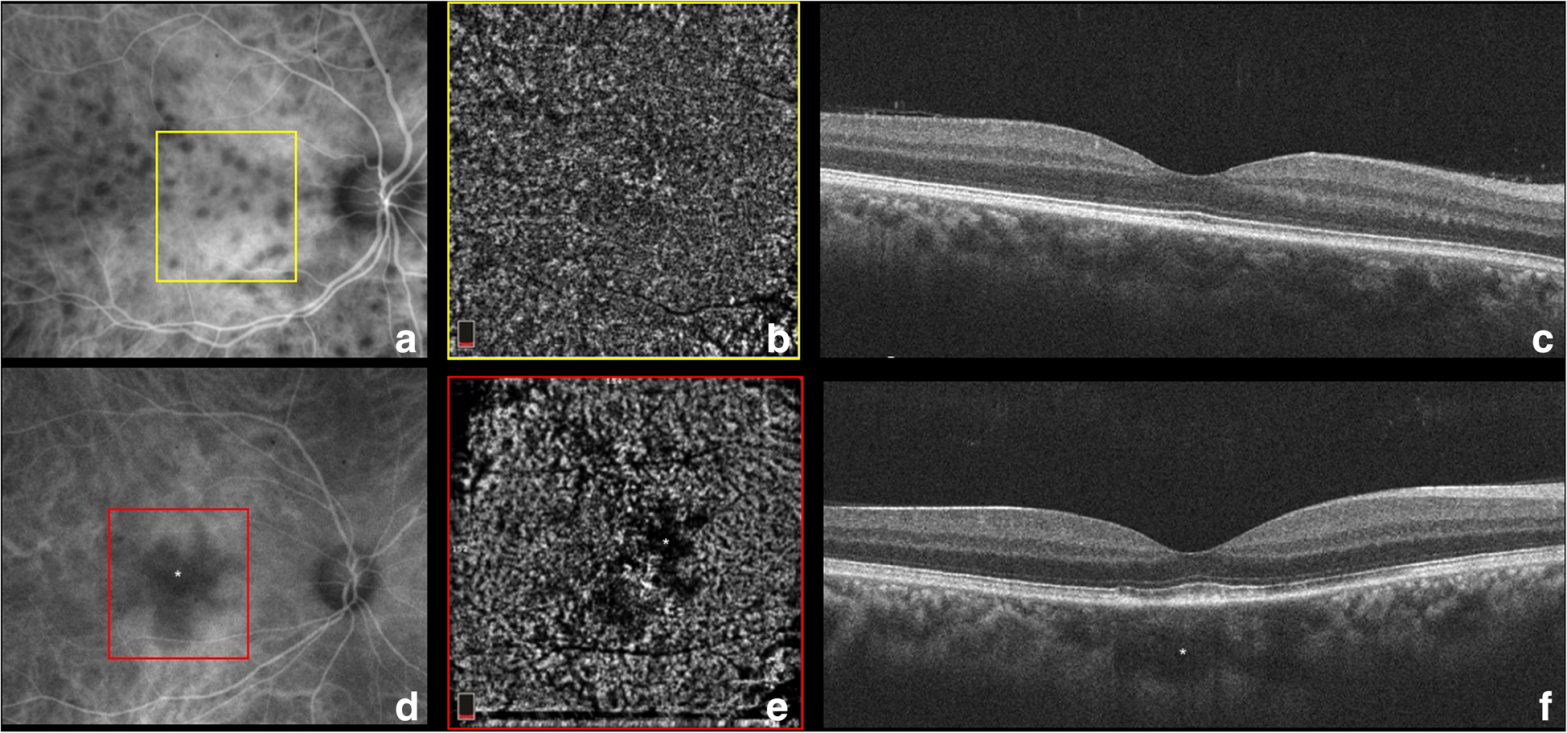
The upper panels (**a**, **b**, **c**) illustrate the right eye of a patient with small sarcoid choroidal granulomas that appear as punctate hypofluorescent lesions with indocyanine green angiography (**a**). These small granulomas are not detected with OCTA at the level of the choriocapillaris (**b**) or by enhanced depth OCT (**c**). This may be because smaller granulomas initially grow in the loose connective stroma of the choroid without alteration of the vascular structure. Their diameter is approximately the size of a choriocapillaris lobule and may not be resolvable with spectral domain OCT. With progressive granuloma growth deeper into the loose choroidal connective tissue, the surrounding vasculature may be shifted and compressed, as illustrated in the bottom panels of the second case. Larger choroidal granulomas tend to occupy the full-thickness of the choroid as noted by the central choroidal hyporeflectance with EDI-OCT (**f**). This lesion can be visualized with OCTA as a central area of choriocapillaris blockage or non-flow (**e**) that colocalizes with ICG hypofluorescence (**d**)

In secondary stromal choroiditis, such as sarcoidosis and TB, the hypocyanescent lesions are distributed more irregularly [18]. Small sarcoid granulomas tend to be localized to the inner choroidal stroma (since leukocyte diapedesis starts in Sattler’s layer where blood vessels have thinner walls [32]) and demonstrate punctate hypofluorescence with ICGA. Since their diameter approximately corresponds to the size of the choriocapillaris lobules and the inflammatory cells initially expand by infiltrating the loose connective tissue between the vascular structures [33], small granulomas are not identified with OCTA (Fig. 5) [3]. As the granulomas grow, they extend in the loose choroidal connective tissue and shift and may compress the surrounding vasculature [34]. Larger, full-thickness choroidal granulomas can be visualized on OCT angiography (Fig. 5) as areas of choriocapillaris non-flow that co-localize with the ICG hypofluorescent lesions [3].

**Fig. 6:**
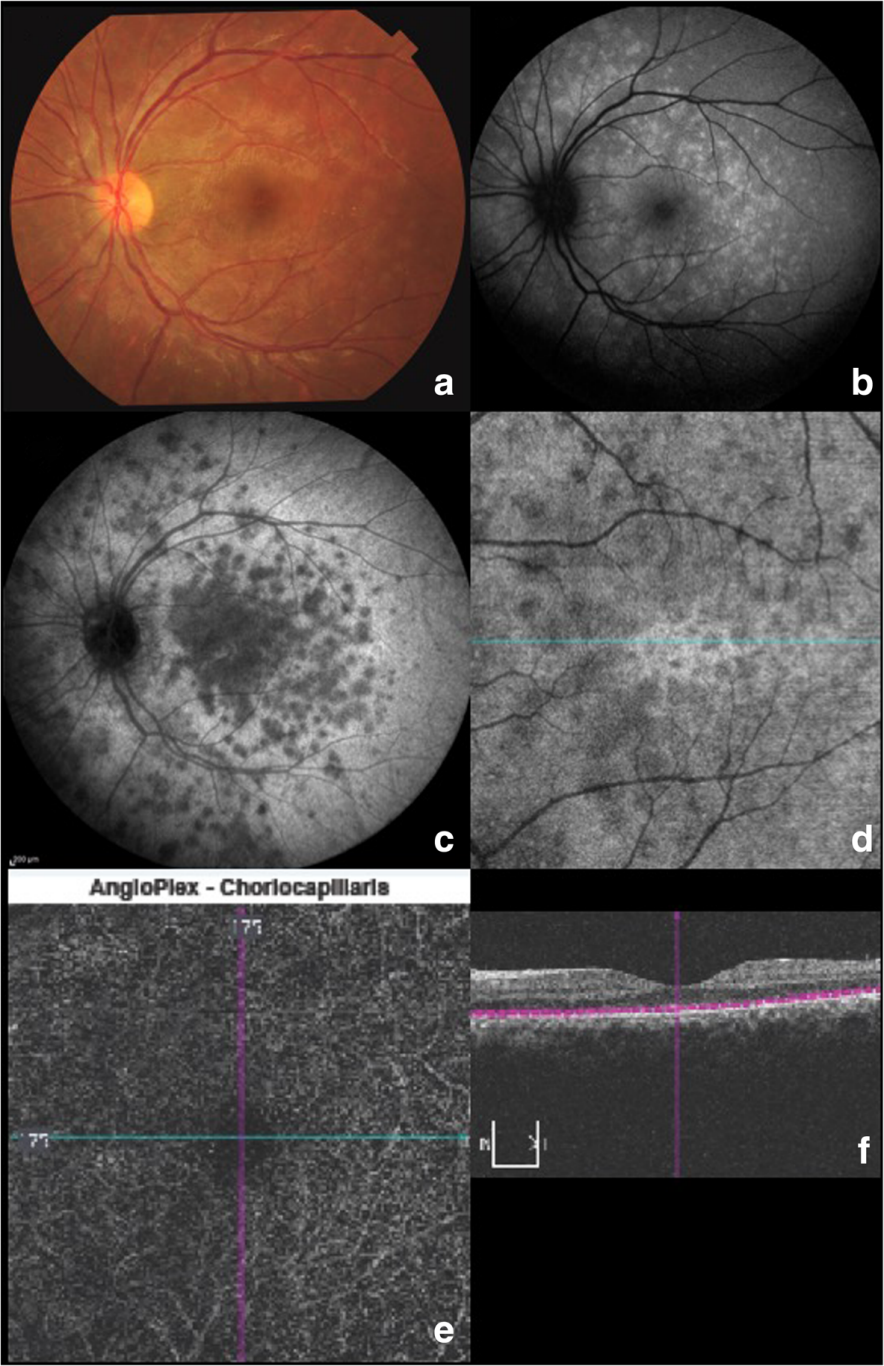
Left eye of a patient illustrating the discrete white retinal lesions or “spots” of MEWDS (**a**). FAF (**b**) highlights the hyper-autofluorescence of the “spots” due to unmasking of the underlying RPE autofluorescence as a result of photoreceptor loss. ICGA illustrates hypofluorescence of these lesions (**c**). This is due to poor absorption of ICG by a diseased RPE-photoreceptor complex, not the result of choroidal ischemia. This is confirmed by the en face OCT (**d**) that illustrates hyporeflectance of the “spots” at the RPE-photoreceptor level, and by OCTA (**e**) of the choroid that does not display any evidence of choriocapillaris ischemia (**f**)

A third, less-known mechanism of hypofluorescence in uveitis has been described by Chang et al. [35] who studied the ICG absorption characteristics of the RPE and noted that the RPE normally absorbs ICG leading to the physiological background hyperfluorescence normally seen with ICGA. ICG uptake, however, may be disrupted in certain retinal disorders such as multiple evanescent white dot syndrome (MEWDS) first reported by Jampol et al. in 1984 [36]. From the early descriptions, electroretinography [37] analysis demonstrated photoreceptor dysfunction that was initially attributed to a primary RPE abnormality, since the hyperfluorescent FA pattern in MEWDS was interpreted as an RPE window defect [38]. With the widespread clinical use of ICGA in the 1990s, the observation of late-phase ICG hypofluorescence suggested a choroidal origin of MEWDS lesions resulting from choroidal hypoperfusion [39]. The “choroidal” lesions were thought to perturb the RPE and outer retina sufficiently to account for the characteristic white spots. Recently, OCTA [40] has for the first time demonstrated completely normal choriocapillaris flow with no evidence of microvascular disruption even in the areas with corresponding ICG hypofluorescence (Fig. 6). On the contrary, the hypofluorescent areas on ICGA co-localize with areas of hyporeflectivity with *en face* OCT at the level of the RPE-photoreceptor complex, thus confirming that the “sick” and mottled RPE cells in these areas do not absorb the ICG molecule leading to dark areas. OCTA has been essential in supporting for the first time the hypothesis that the choriocapillaris is not involved in MEWDS.

### Fundus autofluorescence and optical coherence tomography angiography

Measurement of fundus autofluorescence is a fast and easy non-invasive procedure. A confocal scanning laser employs a band-pass excitation filter at 488 nm and a barrier emission filter above 500 nm. The most significant strength of fundus autofluorescence (FAF) imaging is the capability to assess the integrity of the RPE/photoreceptor complex [41]. One of the most important roles of the RPE is to digest, by lysosomal action, the tips of the outer segments of the photoreceptors that are phagocytosed on a daily diurnal basis. A fraction of these phagocytosed outer segments is chemically incompatible for degradation and therefore accumulates in lysosomes of the RPE as lipofuscin. Accumulation of fluorescent material in the RPE reflects the level of metabolic activity, which is largely determined by the quantity of photoreceptor outer segment turnover.

Integrating OCTA findings in uveitis with FAF may pose various challenges. Abnormally high levels of FAF at the edge of inflammatory lesions may be the result of an abnormal metabolic load that cannot be properly processed by the RPE. In this case, no vascular abnormality is expected and OCTA will be unremarkable. These are typical findings of acute zonal occult outer retinopathy (AZOOR) [42], a nebulous disease that causes visual impairment due to progressive photoreceptor loss. The RPE is sequentially affected following the death of photoreceptor cells. RPE autofluorescence has been employed as a marker for AZOOR activity [43]. In FAF images, hyperautofluorescence at the border of the expanding lesion may be the result of accumulated lipofuscin in RPE cells due to metabolic RPE over-activity related to increased photoreceptor outer segment turnover. Alternatively, loss of photoreceptors may lead to unmasking of the underlying normal RPE autofluorescence [44, 45].

**Fig. 7:**
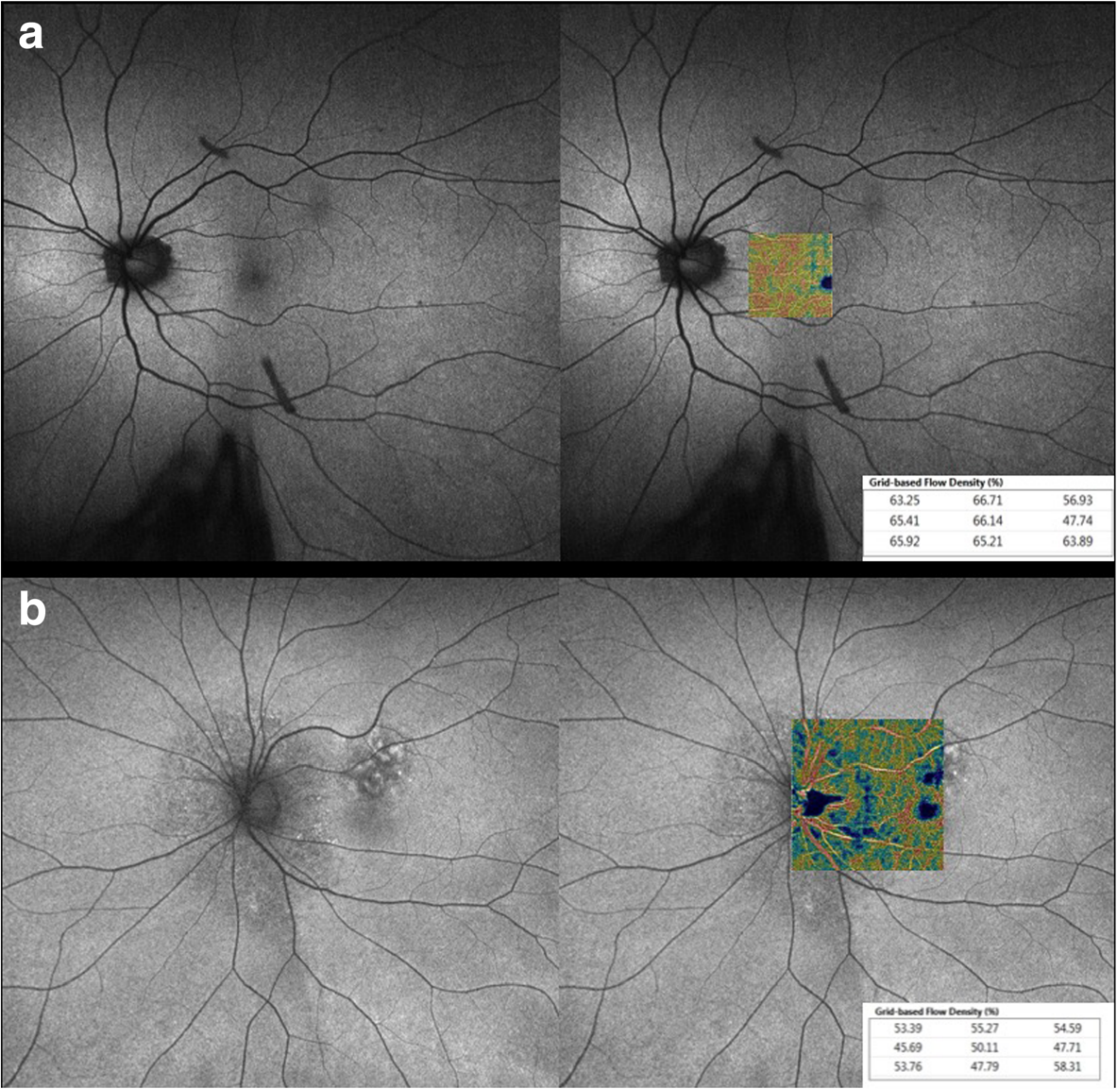
Fundus autofluorescence of the left eye of a patient with active AZOOR (**a**) illustrates hyper-autofluorescence around the disc. The corresponding deep plexus OCT angiogram displays an increased flow corresponding to the active hyper-autofluorescent lesion. A second patient with AZOOR and no activity with fundus FAF (**b**). The color-coded map of the deep capillary plexus on OCTA is superimposed over the FAF image and does demonstrate reduced deep capillary flow at the edge of the inactive AZOOR lesion

Even if OCTA does not possess the capability to detect disruption of the RPE secondary to active inflammation, it may provide other meaningful information. The outer retinal photoreceptors are physiologically maintained by the diffusion of oxygen from the choriocapillaris (80%) below and from the deep retinal capillary plexus (20%) above. In animal models, retinal degenerations leading to loss of photoreceptors have been associated with the disproportionate diffusion of oxygen (from the deep plexus and the choroid) to the middle and inner retina, causing oxidative damage. As such, the deep retinal capillary plexus may be primarily involved with the active stage of AZOOR, leading to outer retinal injury and photoreceptor loss. A future application of OCTA (Fig. 7) will be used to study the deep capillary plexus at the border of the active/inactive AZOOR lesion (as distinguished by FAF). OCTA may illustrate an increase in the deep flow at the level of the AZOOR lesion, which may be the source of damaging mediators of inflammation.

### Enhanced depth imaging and optical coherence tomography angiography

Enhanced depth imaging-OCT (EDI-OCT) provides information on the structure of the choroid [46] by posteriorly displacing the zero-delay point, which is the point of maximal OCT signal sensitivity. Positioning the zero-delay point closer to the choroid, rather than the inner retinal layers, results in an enhanced visualization of the choroid. Compared to ICGA, EDI-OCT has the advantage of providing depth-resolved information [47]. A further advantage is the possibility to obtain cross-sectional images of the choroid that provide the capability to compare the choriocapillaris and choroid using OCTA and EDI-OCT.

In serpiginous choroiditis and serpiginous-like choroiditis [25] and in VKH [31], OCTA of the choriocapillaris layer (below the RPE-Bruch’s membrane complex) illustrates multiple dark foci of flow deficit suggesting choriocapillaris loss or hypoperfusion. The edges of these areas of flow deficit are discrete and clearly demarcated with OCTA and correspond or co-localize with areas of choriocapillaris thickening and hyporeflectivity (indicative of choroidal ischemia on EDI-OCT) [48].

EDI-OCT is also able to detect space-occupying lesions, such as choroidal granulomas [34] (Fig. 5), that can be identified as areas of increased homogeneity (with internal hyporeflectivity) within the choroid with loss of the typical choroidal vascular pattern and that are associated with increased signal transmission effect (unlike large choroidal vessels). As previously described, small choroidal granulomas associated with sarcoidosis or TB is localized in the inner choroidal stroma and can be demonstrated by EDI-OCT but not identified with OCTA (Fig. 5c). As the granulomas grow, they extend in the loose choroidal connective tissue and shift and compress the surrounding vasculature. Larger, full-thickness choroidal granulomas can be visualized on OCTA as areas of choriocapillaris non-flow that co-localize with hyporeflective lesions on EDI-OCT (Fig. 5f).

## Conclusions

Understanding the various findings on OCTA in patients with uveitis is an important step towards integrating this advanced novel technology in the routine clinical environment. OCTA may be best employed in a multimodal approach in conjunction with other integral retinal imaging systems. To optimally interpret changes in the vascular flow detected by OCTA, multimodal imaging is still essential. By comparing findings on OCTA with data obtained from traditional systems including dye-based angiographic devices (e.g., wide-field FA and ICGA) and FAF and spectral domain OCT, we are gaining essential information on the pathogenesis of various inflammatory conditions, developing more optimal and reliable follow-up protocols in a non-invasive way, and more accurately and objectively assessing the response to treatment in uveitis patients.

## Abbreviations

APMPPE: Acute posterior multifocal placoid pigment epitheliopathy
AZOOR: Acute zonal occult outer retinopathy
CNV: Choroidal neovascular membrane
EDI: Enhanced depth imaging
FA: Fluorescein angiography
FAF: Fundus autofluorescence
ICGA: Indocyanine green angiography
MEWDS: Multiple evanescent white dot syndrome
OCT: Optical coherence tomography
OCTA: Optical coherence tomography angiography
RPE: Retinal pigment epithelium
TB: Tuberculosis
VKH: Vogt-Koyanagi-Harada
